# Syndecans and Enzymes for Heparan Sulfate Biosynthesis and Modification Differentially Correlate With Presence of Inflammatory Infiltrate in Periodontitis

**DOI:** 10.3389/fphys.2019.01248

**Published:** 2019-09-25

**Authors:** Roko Duplancic, Marija Roguljic, Ivan Puhar, Nika Vecek, Ruzica Dragun, Katarina Vukojevic, Mirna Saraga-Babic, Darko Kero

**Affiliations:** ^1^Study Programme of Dental Medicine, School of Medicine, University of Split, Split, Croatia; ^2^Department of Oral Pathology and Periodontology, Study Programme of Dental Medicine, School of Medicine, University of Split, Split, Croatia; ^3^Department of Periodontology, School of Dental Medicine, University of Zagreb, Zagreb, Croatia; ^4^Department of Anatomy, Histology and Embryology, School of Medicine, University of Split, Split, Croatia; ^5^Laboratory for Early Human Development, School of Medicine, University of Split, Split, Croatia

**Keywords:** periodontitis, syndecans, heparan sulfate, EXTs, NDSTs, inflammatory response

## Abstract

Periodontitis is a common degenerative disease initiated by the bacteria in subgingival biofilm. The exposure to bacterial biofilm triggers host inflammatory response whose dysregulation is ultimately responsible for the destruction of hard and soft periodontal tissues resulting in tooth loss. To date, significant effort has been invested in the research of the involvement of host cells and inflammatory mediators in regulation of inflammatory response in periodontitis. Syndecans (Sdcs) belong to a four-member family of heparan sulfate proteoglycans (HSPGs). Sdcs are compound molecules comprised of the core protein to which several heparan sulfate (HS) glycosaminoglycan (GAG) chains are attached. The role of Sdcs in pathogenesis of periodontitis is poorly investigated despite the numerous reports from experimental studies about the critical involvement of these factors in modulation of various aspects of inflammatory response, such as the formation of inflammatory mediators gradients, leukocyte recruitment and extracellular matrix remodeling in resolution of inflammation. Most of these functions of Sdcs are HS-related and, thus, dependent upon the structure of HS. This, in turn, is determined by the combinatorial action of enzymes for biosynthesis and modification of HS such as exostosis (EXTs), sulfotransferases (NDSTs), and heparanase 1 (HPSE1). The data presented in this study clearly indicate that some Sdcs display different expression profiles in healthy and diseased periodontal tissue. Additionally, the differences in expression profiles of HS GAG biosynthesis and modification enzymes (EXTs, NDSTs, and HPSE1) in healthy and diseased periodontal tissue imply that changes in HS GAG content and structure might also take place during periodontitis. Most notably, expression profiles of Sdcs, EXTs, NDSTs, and HPSE1 differentially correlate with the presence of inflammatory infiltrate in healthy and diseased periodontal tissue, which might imply that these factors could also be involved in modulation of inflammatory response in periodontitis.

## Introduction

Periodontitis is a chronic disease which affects tooth supporting tissue (gingiva, alveolar bone, periodontal ligament) causing its gradual degradation to the point where masticatory function of the teeth (or their resilience to occlusal forces) is permanently lost. The etiology of periodontitis is multifactorial – susceptibility to periodontitis can be associated to various demographic factors (age, sex, social background), micro-environmental factors (chronic bacterial and endotoxin exposure from subgingival microbial biofilm), and/or constitutive host traits (both genetic and epigenetic). To a variable degree, all these factors can modulate host inflammatory response, which (if dysregulated) causes progressive destruction of periodontal tissue (Hernandez et al., 2011; Silva et al., 2015).

Syndecans (Sdcs) belong to a four-member family (Sdc1-4) of cell surface heparan sulfate proteoglycans (HSPGs). Sdcs are compound molecules comprised of a cell membrane embedded core protein (divided into cytoplasmic, transmembrane, and ectoplasmic domains) to which several heparan sulfate (HS) glycosaminoglycan (GAG) linear side chains are covalently attached (Bernfield et al., 1992; Gotte, 2003; Multhaupt et al., 2009). With regard to functional versatility of Sdcs and, consequently, their roles in regulation of inflammatory response, there are several important aspects which need to be considered, some of which are more exclusive to Sdcs, and some of which can be broadly associated to HSPGs. In adult tissues, Sdcs are widely distributed, however, the expression domains of individual Sdcs do not completely overlap – Sdc1 is predominantly expressed in stratified and simple epithelia, Sdc2 in connective tissue and endothelial cells, Sdc3 in neural tissue, whereas Sdc4 is expressed ubiquitously. The expression of Sdcs can be very responsive to changes in micro-environment, and this is especially the case in developing tissues during the early stages of organogenesis when expression patterns of Sdcs can significantly deviate from the patterns observed in adult tissues (Vukojevic et al., 2012; Filatova et al., 2014; Kero et al., 2015; Cubela et al., 2016). Studies on *Sdc1^–/–^* and *Sdc4^–/–^* single knockout mice have revealed that these animals develop normally, meaning that Sdc1 and Sdc4 are functionally redundant in organogenesis. However, when adult *Sdc1^–/–^* or *Sdc4^–/–^* knockouts are challenged by disease-instigating agents they, depending on the type of challenge, display impaired regulation of various segments of inflammatory response – from formation of chemokine/cytokine gradients and leukocyte recruitment, to ECM remodeling in resolution of inflammatory response (Floer et al., 2010; Teng et al., 2012).

Generally, biochemical properties enable HSPGs to bind various factors, and while this binding can be mediated via specific sequences in core protein domains, interactions with extracellular matrix (ECM) components, growth factors, morphogens, and mediators of disease pathogenesis are mostly HS-related (Taylor and Gallo, 2006; Roper et al., 2012; Stepp et al., 2015). Biochemical properties of HS greatly depend on HS chain length and composition (the latter is also determined by adding sulfate groups in a specific pattern). HS can even be post-biosynthetically cleaved into short fragments by enzymes such as heparanase 1 (HPSE1) whose activity is reportedly elevated in conditions such as inflammation or malignant alteration (Li and Vlodavsky, 2009; Stoler-Barak et al., 2014; Gutter-Kapon et al., 2016). The framework for HS biosynthesis is laid down by the combinatorial action of several enzymes including exostosin-glycosyltransferases (Exts/EXTs) (chain elongation) and bi-functional sulfotrasferases (Ndsts/NDSTs) (initial sulfation modifications) (Esko and Selleck, 2002; Presto et al., 2008). Although the non-redundancy of particular Exts/EXTs and/or Ndsts/NDSTs in organogenesis has been long established, recent studies on targeted endothelial ablation of various genes encoding for HS biosynthesis enzymes show that they are also critical for regulation of inflammatory response, pretty much in the same manner as previously described for Sdcs in challenged *Sdc1^–/–^* and *Sdc4^–/–^* knockouts (Poon et al., 2014; Ge et al., 2018; Talsma et al., 2018).

There are only few studies about the possible involvement of Sdcs in pathogenesis of periodontitis in humans (Oksala et al., 1997; Manakil et al., 2001; Kotsovilis et al., 2010). While the differential expression of several HSPGs (Sdc1, CD44, decorin, and biglycan) between healthy and diseased periodontal tissue was reported, there were no data about other functionally related factors to HSPGs, including the enzymes for biosynthesis and modification of HS. Furthermore, due to less advanced imaging techniques and methods for quantification of immunohistochemical staining, no adequate correlation of observed expression patterns with various histomorphometric parameters relevant for the subject could be done. Therefore, we sought to provide more detailed description of expression patterns of Sdcs and enzymes for biosynthesis and modification of HS in healthy and diseased human periodontal tissue, as well as to discuss our findings in light of the important roles generally attributed to HSPGs in regulation of inflammatory response.

## Materials and Methods

### Participants Screening and Recruitment

40 patients were referred from their appointed general practitioners to the Department of Oral Pathology and Periodontology, University of Split Hospital Center, and the Department of Periodontology, University of Zagreb Hospital Center. Standard protocol was used to determine diagnosis including detailed medical history, radiographic examination and periodontal status. Periodontal status was assessed by two experienced and calibrated dental examiners who recorded probing depth (PD), gingival recession (RE), clinical attachment level (CAL), full-mouth plaque (FMPS), and bleeding (FMBS) scores. Clinical measurements were taken at six sites per tooth using a standard periodontal probe (PCP 15; Hu-Friedy, Chicago, IL, United States). The diagnosis was established according to new classification of periodontal and implant diseases and conditions (Papapanou et al., 2018). Participants were informed about the purpose of this study and clinical procedures related to it, and gave their written consent.

The inclusion criteria were: age of at least 18 years, good general health (no systemic diseases), healthy periodontal tissue (controls) and generalized periodontitis stage III or IV (periodontitis group) according to guidelines for classification of periodotal and peri-implant diseases (Papapanou et al., 2018). The exclusion criteria were: presence of periodontal abscess and endo-periodontal lesions in the vicinity of the sampling area, systemic conditions affecting periodontal tissue (e.g., diabetes mellitus), long-term medication, medical history of systemic antibiotic therapy within the last 6 months, pregnancy, alcohol, or drug abuse. Participants were divided into two groups: subjects with healthy periodontal tissue (controls; *n* = 20), and subjects with untreated generalized periodontitis (stage III or IV) having at least one tooth indicated for extraction due to poor condition of periodontal tissue (periodontitis group; *n* = 20). Group demographics and clinical parameters are listed in Table 1.

**TABLE 1:** Demographic and clinical parameters of participants from control and periodontitis group.

	**Controls (*n* = 20)**	**Periodontitis (*n* = 20)**
Age^∗∗∗^ (years)	38,9 ± 9,23	50,26 ± 10,6
Sex (F/M)	8 F/12 M	9 F/11 M
Smokers^∗∗∗^	No	17
FMPS^∗^ (%)	21,95 ± 8,95	36,17 ± 15,76
FMBS^∗∗∗^ (%)	8,95 ± 3,73	43 ± 11,27
PD^∗∗∗^ (mm)	1,47 ± 0,22	4,53 ± 1,19
CAL^∗∗∗^ (mm)	1,52 ± 0,22	6,1 ± 1,43

*FMPS, Full Mouth Plaque Score; FMBS, Full mouth bleeding score; PD, Probing depth; CAL, Clinical attachment level. Level of significance was set at α = 0,01 (^∗∗∗^*p* < 0,01; F_*crit*_ = 7,325545); (^∗^border values, level of significance α = 0,05); Statistically significant difference between the groups was found for age (*p* = 0,007571), smoking (*p* = 0,000001), FMPS (*p* = 0,001056), FMBS (*p* = 0,000216), PD (*p* = 0,000579), and CAL (*p* = 0,000631). No statistically significant difference was found for sex distribution between groups (*p* = 1).*

Participants with periodontitis received initial periodontal therapy (1 visit per quadrant) in period of 7 days by two experienced periodontists. In the initial visit, a single tooth was extracted using periotome (Aesculap AG, Tuttlingen, Germany) for atraumatic extraction, The sample of diseased gingiva (from gingival margin to the edge of residual alveolar bone) was taken with a type 15C scalpel blade (Aesculap AG, Tuttlingen, Germany) on the buccal aspect of tooth extraction site (average sample dimensions 4 mm × 2,5 mm). Extraction sites were sutured with monofilament sutures (Péters Surgical, Bobigny, France).

Participants from control group were initially referred for crown lengthening with regard to preparation for prosthetic therapy either by general practitioners or prosthodontists. Surgical crown lengthening was performed under local anesthesia and included external gingivectomy (used for the sampling of healthy gingiva), as well as osteotomy and osteoplasty. The samples of healthy gingiva (average dimensions 2,5 mm × 1,5 mm) were taken from the buccal aspect of gingival margin down to the alveolar bone margin (before the osteotomy and osteoplasty were performed). Sampling sites were sutured with monofilament sutures (Péters Surgical, Bobigny, France). After the samples were taken, they were stored in sealed containers with paraformaldehyde and delivered to laboratory within 24 h. The follow-up of participants from both groups adhered to established guidelines for the type of treatment they received.

### Human Gingival Samples Procurement and Processing

For this study, the total of 40 samples of human gingiva (20 per group) were analyzed. Tissue procurement and processing were approved by the Ethical and Drug Committee of School of Medicine, University of Split (Class: 003-08/17-03/0001, No: 2181-198-03-04-17-0043) and Ethical and Drug Committee of School of Dental Medicine, University of Zagreb (No: 05-PA-15-6/2017) in accordance with Helsinki Declaration (Williams, 2008). Before the fixation in 4% paraformaldehyde, vestibular (labial/buccal) surfaces of gingival samples were marked by waterproof color in order to facilitate proper orientation of the samples during paraffin embedding. The samples were cut in serial 5 μm thick sections and mounted on glass slides. Preservation of the tissue and positioning of the structures of interest were examined on orientation slides stained with hematoxylin and eosin (H/E) (every 10th slide in each sample). After that, part of the samples was stored in the archival collection of the Department of Anatomy, Histology and Embryology (School of Medicine, University of Split) and designated with unique depersonalized codes to hide the identity of sample donors, and the rest was used for immunofluorescence staining.

### Immunofluorescence Staining

Deparaffinization and staining protocols were previously described (Kero et al., 2016). We slightly modified the staining protocol by introduction of protein blocking (ab64226; Abcam, United Kingdom) for 30 min before the application of primary antibodies. The following primary antibodies were used: mouse monoclonal anti-Sdc1 [B-A38] (1:100, ab34164, Abcam, United Kingdom), rabbit polyclonal anti-Sdc2 (1:200, ab191062, Abcam, United Kingdom), rabbit polyclonal anti-Sdc4 (1:100, ab24511, Abcam, United Kingdom), rabbit polyclonal anti-EXT1 (1:100, ab126305, Abcam, United Kingdom), rabbit polyclonal anti-EXT2 (1:50, ab102843, Abcam, United Kingdom), rabbit polyclonal anti-NDST1 (1:50, Abcam, United Kingdom), rabbit polyclonal anti-NDST2 (1:100, ab1511141, Abcam, United Kingdom), rabbit polyclonal anti-HPSE1 (1:200, ab85543, Abcam, United Kingdom) and mouse monoclonal anti-CD45 [MEM-28] (1:200, ab8216, Abcam, United Kingdom) as a general inflammatory cell marker (Wu et al., 2018). Secondary antibodies were used at 1:400 dilution: anti-mouse Alexa Fluor 488 (GREEN, ab150105, Abcam, United Kingdom), anti-rabbit Alexa Fluor 594 (RED, ab150092, Abcam, United Kingdom), and anti-rabbit Alexa Fluor 488 (GREEN, ab150077, Abcam, United Kingdom). To stain cell nuclei, sections were shortly incubated with 4′6′-diamidino-2-phenylindole (DAPI). Samples of oral mucosa from maxillary tuberosity were stained for positive control (total of 30 sections on 15 slides). Both single and double immunofluorescence staining was performed. For double immunofluorescence control, mouse anti-Sdc1 and anti-CD45 were stained in tandem with the rest of the primary antibodies (all rabbit polyclonal) and compared with single immunofluorescence staining. Expression patterns (nuclear vs. non-nuclear) were analyzed by intensity correlation analysis of photo-micrographs at magnifications ×20 and ×40 in ImageJ (NIH Public Domain, Bethesda, MD, United States) according to previously described method (Kero et al., 2017). Color scatter-plots for all primary antibodies displayed non-nuclear expression pattern (data not shown).

### Acquisition and Processing of H/E Panoramic Images

H/E stained slides were examined under Olympus BX40 (Olympus, Tokyo, Japan) microscope equipped with manual slide scanner (Microvisioneer, Esslingen am Necker, Germany), standard (Olympus DP27, Olympus, Japan) and area-scan high-resolution camera (Basler aceA2500-14gm, Basler, Germany). H/E panoramic images were captured at magnification ×20 (camera settings – exposure time: 8 ms; ISO: 100). Panoramic images were exported and processed in Adobe Photoshop^®^ CC (2014). Rotation of panoramic images was done using 40 customized presets (one for each sample), and high resolution background (600 dpi) was used for image size reduction in order to minimalize image detail loss. Additionally, regions of epithelial tissue on each panoramic image were masked using graphic pen tablet (Wacom Intuos PRO, Wacom Co, Saitama, Japan). Total section area and areas of specific tissue compartments (epithelia, subepithelial stroma) were then measured in Adobe Photoshop^®^ CC and expressed in pixels (px).

### Acquisition and Processing of Panoramic Immunofluorescence Images

Slides stained for immunofluorescence were examined at ×10 magnification using Zeiss Axio Observer (Carl Zeiss Microscopy GmbH, Jena, Germany) equipped with Zeiss Axiocam 506 color camera (Carl Zeiss Microscopy GmbH, Jena, Germany) set for full frame resolution (2752 × 2208 px) in original black and white and 8-bit depth (256 px values) pseudo-colorizer mode. Acquisition and merging of photo-micrographs into panoramic immunofluorescence (IF) images were done using multichannel and panorama modules in ZEN 2.5 software (Carl Zeiss Microscopy GmbH, Jena, Germany), respectively. After background calibration (0 px), exposure time in multichannel module was set at 35 ms for DAPI. For green (AF488) and red (AF594) fluorescence, exposure time was additionally calibrated after signal output equalization at 800 ms (green) and 12000 ms (red) (green and red fluorescence to Sdc1 used as reference). For the stitching of photo-micrographs (tiles) into panoramic IF images, tiles were captured sequentially by manual navigation along x/y axes guided by the on-screen live view of DAPI channel with minimal 20% overlap (x) and 10% maximal shift (y) between individual tiles. Panoramic IF images were closely inspected for quality (tiles alignment, absence of stitching artifacts) and stored in raw format (CZI – Zeiss proprietary file format) containing original data and metadata. Each file was then exported in multiple TIFF format files (original black and white, pseudo-colorized and merged files). Black and white images were subsequently processed for quantification of staining. Rotation and masking were done in a similar manner described for panoramic H/E images, although more restrictive procedure for image size reduction (300 dpi backgrounds) had to be applied in order to format files for analysis in ImageJ. For the calculation of total section area, merged IF panoramic images (factor/DAPI) were thresholded at 1 px (cut-off threshold was set at 10 px). In order to measure the area of epithelial and subepithelial tissue compartments, images were merged with customized black and white masks using “darken image” blending mode in Adobe Photoshop^®^ CC. Thus, for each panoramic IF image, five additional images were produced (threshold area, mask, inverted mask, epithelial, stromal). All area measurements were subsequently used as reference values for the calculation of expression domain size (cumulative and fractional output) so they could be expressed in percentages (%).

### Intensity Distribution – Histograms and Heatmaps

Histograms for panoramic IF images were made in ImageJ and exported as tables where for each x value (intensity in px from 0 to 255 px) a y-value (number of points with corresponding intensity) is listed. The tables were then used to calculate the size of expression domains (cumulative and fractional values expressed in%) in individual samples and as group means. Since we set the cut-off at 10 px, each table contained 245 values. Tables with cumulative and fractional values were also used for statistical analysis. The protocol for multi-color heatmaps was described previously (Kero et al., 2018). Here, we have only slightly modified the increments of intensity range in order to accommodate for four-color heatmaps as follows: blue (10–60 px), green (60–150 px), red (150–255 px), and yellow (255 px).

### Densitometry – 2D Plot Profiles and 3D Surface Plots

To represent expression patterns in the form of spatial gradients, 2D plot profiles and 3D surface plots of panoramic IF images were made as described previously (Kero et al., 2018). However, we omitted the conversion of px values to optical density units. For each investigated factor, 2D plot profiles were made in oral-sulcular direction (left-right plot), as well as in marginal-apical direction (top-down plot) (Figure 4). Multiple colocalization of expression patterns of all investigated factors was done by 3D surface plots.

### Statistical Analysis

The following histomorphometric parameters were analyzed for each sample: total section, epithelial and stromal compartment areas, total, epithelial, and stromal celularity (using DAPI nuclear staining) and internuclear space, total and stromal distribution of inflammatory infiltrate (using CD45 staining), and total, epithelial and stromal areas of expression domains of investigated factors. In both groups, a group mean of the area of expression domains of each investigated factor was calculated (represented in percentages as fractional values on 10–255 px scale) for total section and tissue compartments (epithelial/stromal). Cumulative values of histomorphometric parameters were presented by column graphs. Single factor ANOVA was used for comparison of expression domains between control and test group. Correlation of expression domains was done by multiple linear regression. In order to reduce bias of statistical analyses of histomorphometric parameters and expression patterns of investigated factors, sections from both groups were divided into two major tissue compartments – epithelial compartment and subepithelial stroma compartment. The epithelial compartment included gingival oral epithelium, sulcus epithelium and pocket epithelium (when preserved in samples from periodontitis group). Demographics and clinical parameters of study participants were also analyzed (descriptive statistics, *t*-test for continuous variables and Fisher’s exact test for categorical variables). Statistical tests were performed in Microsoft Office Excel^®^ 2016 (Microsoft Corp., Redmond, WA, United States) and GraphPad v8 (GraphPad Software, La Jolla, CA, United States). Statistical significance (α) was set at 0.01 (*p* < 0.01).

## Results

### Histomorphometry – Tissue Morphology and Celularity

**FIGURE 1:**
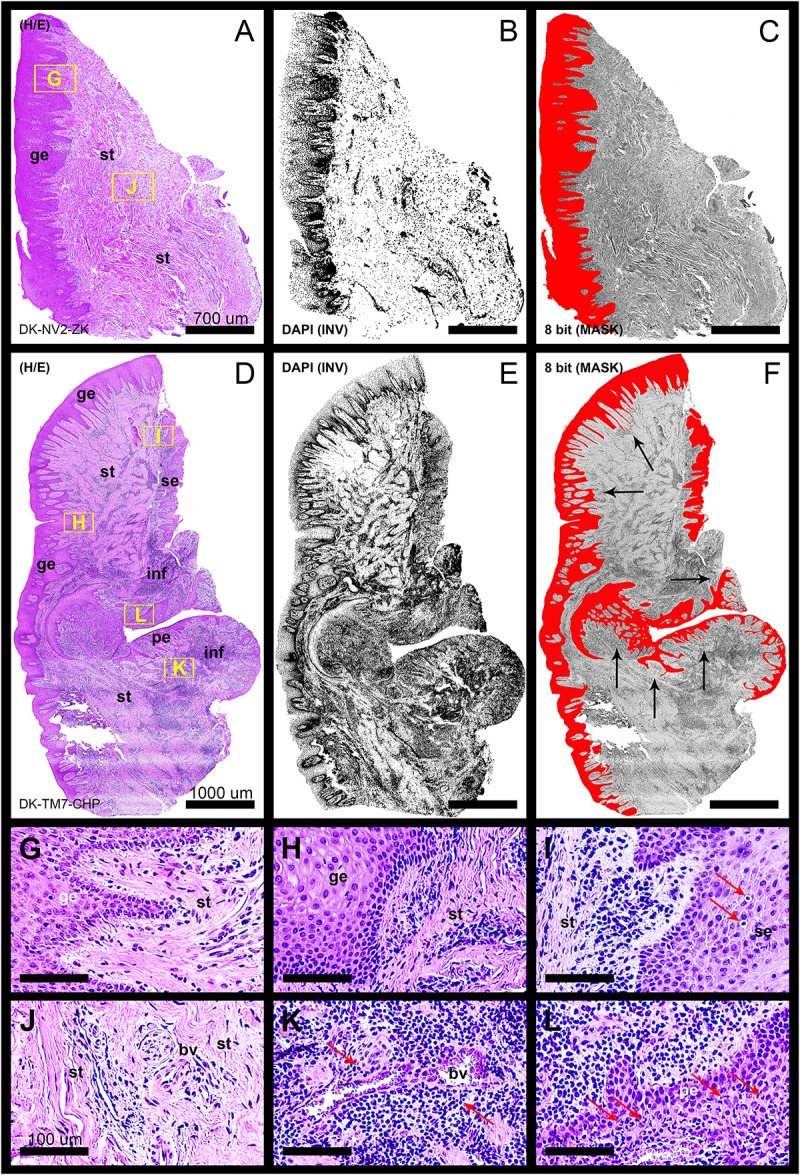
Panoramic H/E images **(A,D)** of healthy (control sample DK-NV2-ZK) and diseased gingiva (periodontitis sample DK-TM7-CHP). In contrast to thickened gingival epithelium **(C)** and clear subepithelial stroma with scarce perivascular inflammatory infiltrate **(B,G,J)** of healthy gingiva, the diseased gingiva displays thinned gingival epithelium with no cytologic atypia but extended rete pegs and features of pseudoepithelial hyperplasia from the pocket epithelium (**F**, black arrows). The abundant inflammatory infiltrate can be seen along the perivascular spaces (**K**, red arrows) and diffusely toward sulcular and apical segments of the gingiva **(E,I,L)** comprised mostly of lymphocytes. Few lymphocytes infiltrating sulcus epithelium are visible (**I**, red arrows) Pocket epithelium is infiltrated by many granulocytes (**L**, red arrows). The difference in total celularity levels between healthy and diseased gingiva are clearly visible on inverted DAPI panoramic images **(B,E)**. Magnification: **(A–L)** ×20; *scale bars –* **(A–C)** 700 μm; **(D–F)** 1000 μm; **(G–L)** 100 μm. Designations : gingival epithelium (ge), subepithelial stroma (st), sulcus epithelium (se), pocket epithelium (pe), inflammatory infiltrate (inf), blood vessel (bv).

**FIGURE 2:**
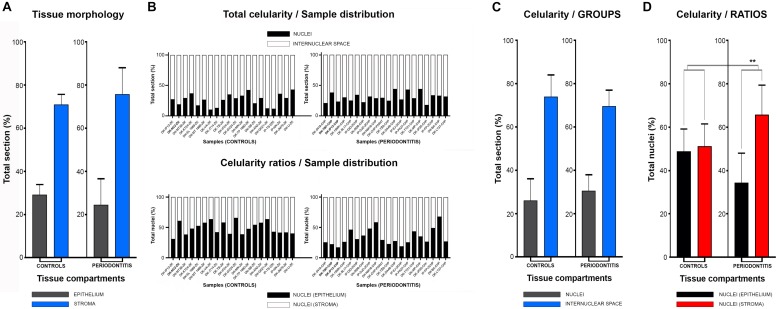
Histomorphometry of gingival samples from control and periodontitis group. Analysis of fractions of epithelial and stromal section compartments (expressed here as group means) showed no statistically significant difference between control and periodontitis group (*p* = 0,63899, α = 0,01) **(A)**, and thus tissue morphology could be excluded as potential bias for subsequent statistical analysis. Total celularity (expressed as fractions of nuclei and internuclear space) and epithelial/stromal celularity ratios (expressed as ratio of total epithelial vs. total stromal nuclei) are presented as whole-in-one columns (for individual samples from both groups) **(B)** and as group mean values **(C,D)**. No difference in total celularity was observed between groups (*p* = 0,12603, α = 0,01) **(C)**. However, the epithelial/stromal nuclei ratios in control samples (1:1) and in periodontitis group (close to 1:2) displayed statistically significant difference (^∗∗∗^*p* = 0,00371, α = 0,01) indicating perturbed homeostasis of gingival tissue in periodontitis.

**FIGURE 3:**
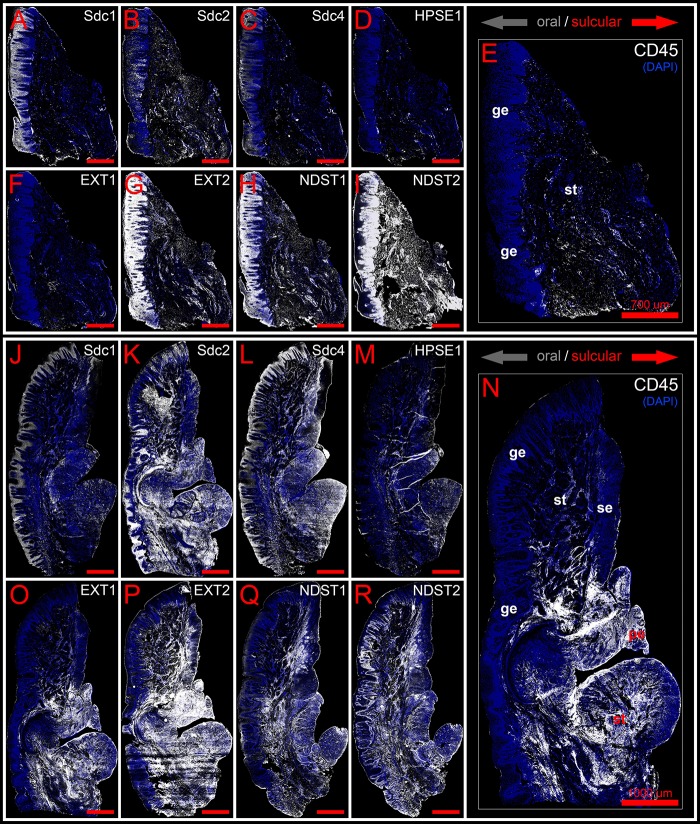
Panoramic IF images of expression of Sdcs, EXTs, NDSTs, and HPSE1 in healthy (control sample DK-NV2-ZK) **(A–I)** and diseased gingiva (periodontitis sample DK-TM7-CHP) **(J–R)** compared with expression of inflammatory marker CD45 **(E,N)**. Fluorescence signals are shown in white color. Panoramic IF images are merged with DAPI nuclear stain (pseudo-colorized in blue). Notice how Sdcs, HPSE1, and EXT1 display altered patterns of stromal expression in healthy **(A–D)** and diseased gingiva **(J–M)**, whereas EXT2 and NDSTs display altered patterns of epithelial expression **(G–I,P–R)**. Magnification: **(A–R)** ×10; *scale bars* – **(A–I)** 700 μm; **(J–R)** 1000 μm. Designations : gingival epithelium (ge), subepithelial stroma (st), sulcus epithelium (se), pocket epithelium (pe). Oral/vestibular aspect of tissue section (gray arrows); Sulcular/pocket aspect of tissue section (red arrows).

In general terms, gingiva is comprised of two major tissue compartments – epithelial tissue (stratified epithelium) and underlying connective tissue (subepithelial stroma). Using the panoramic H/E images (Figures 1A,C,D,F), the area size of epithelial and stromal compartments for each sample was calculated as a fraction of total section and subsequently expressed in percentages as group means. On average, epithelial/stromal fraction for controls was 29,27/70,87%, and for periodontitis group 24,27/75,73% (*p* = 0,63899). No statistically significant difference was found between the groups (Figure 2A) meaning the overall tissue section morphology should not be considered as a confounding factor for further statistical analyses. Total celularity of tissue samples was calculated as a fraction of area covered by DAPI nuclear staining (Figures 1B,E) from the total section area and expressed in percentages as group means. Surprisingly, there was no statistically significant difference between the groups – total nuclei/internuclear space fraction for controls was 22,55/77,45%, and for periodontitis group 28,31/71,69% (*p* = 0,12603) (Figures 2B,C). However, once the celularity was calculated separately for epithelial and stromal compartments, the differences between the groups became apparent. Namely, epithelial/stromal nuclei ratio in controls was 1:1 (49,54/50,46%), whereas in periodontitis group it was almost 1:2 in favor of stromal compartment (34,36/65,54%) (*p* = 0,00371) (Figure 2D). There are couple of possible explanations for such findings. Epithelial/stromal celularity ratio in periodontitis group could be partially attributed to the increased presence of inflammatory cells, which in some cases diffusely infiltrated subepithelial stroma (Figures 1K,L, 3N). The expression of general inflammatory cell marker CD45 was also found to be statistically significantly different between controls and periodontitis group (stromal ratio 1,76/9.47%, *p* = 0.00349) (Figure 7D). It should be noted that in several gingival samples from periodontitis group, the inflammatory cell infiltrate was limited to narrow perivascular spaces in subepithelial stroma, and its amount was on comparable levels to those observed in controls (Figures 1J, 3E, 5B), so it is possible that changes in other cell populations might also be accounted for differences in epithelial/stromal celularity ratios between the groups.

### Expression of Sdcs and HPSE 1 – IHC and Densitometry

**FIGURE 4:**
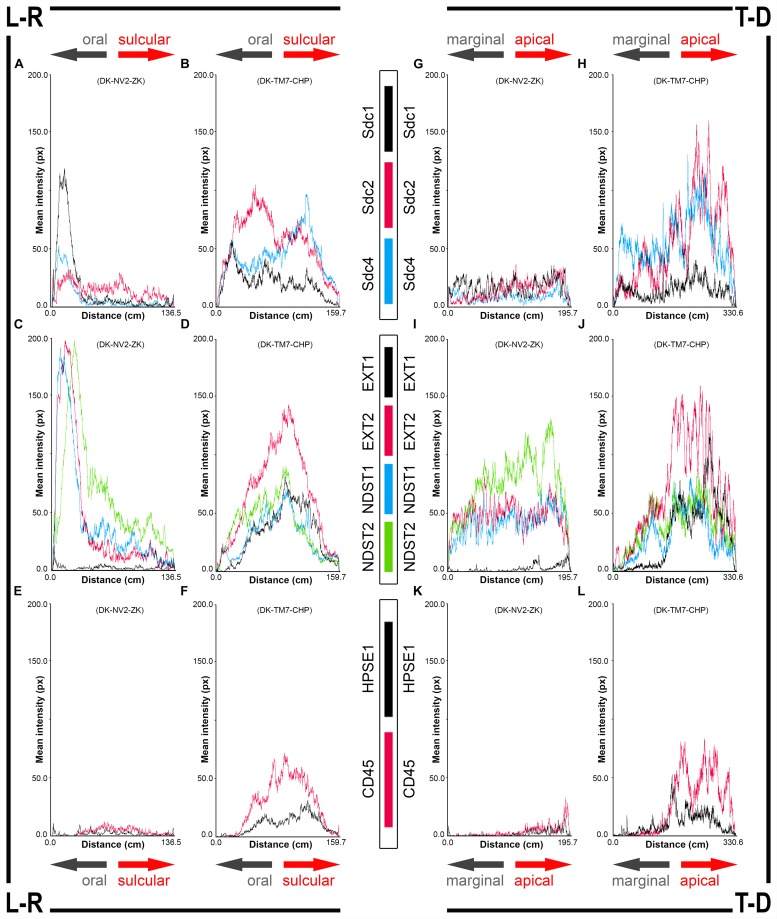
2D plot profiles of investigated factors in healthy **(A,C,E,G,I,K)** (sample DK-NV2-ZK) and diseased gingiva **(B,D,F,H,J,L)** (sample DK-TM7-CHP). Fluorescence signals from panoramic IF images are plotted as curves depicting mean expression intensity from oral toward sulcular aspect (L-R plots) **(A–F)** and from marginal toward apical aspect (T-D plots) **(G–L)** of tissue sections. Note that mean expression intensities of most investigated factors in diseased gingiva display positive trend in sulcular and apical direction. (*x*-axis – scanning distance in cm; *y*-axis – mean intensity in px; arrows point toward specific aspect of tissue section).

In controls, the expression of individual Sdcs could be seen in both gingival epithelium and subepithelial stroma but did not significantly deviate from the previously established pattern – Sdc1 was predominant in epithelial compartment, Sdc2 in stromal compartment, while the expression of Sdc4 was equally balanced between the two tissue compartments but at lower levels than expression of Sdc1 and Sdc2 (Figures 3A–C). Very weak expression of HPSE1 was observed in subepithelial stroma (Figure 3D). 2D plot profiles for Sdcs reveal either decreasing mean expression intensity gradients in oral-sulcular direction, and flat gradients in marginal-apical direction (Figures 4A,G). Low mean expression intensity and flat intensity gradients could be seen for HPSE1 and CD45 in both 2D plot profiles (Figures 4E,K). In periodontitis group, the expression of Sdcs and HPSE1 maintained similar patterns in gingival epithelium as observed in controls, but they were more intensely expressed in subepithelial stroma (Figures 3J–L, 6A,B,D,E). Consequently, changes in mean intensity levels and gradients could be observed in both types of 2D plot profiles (Figures 4B,H,F,L, 6C,F) showing increasing trend toward sulcular and apical portions of subepithelial stroma where more intense expression of inflammatory marker CD45 was also observed.

**FIGURE 5:**
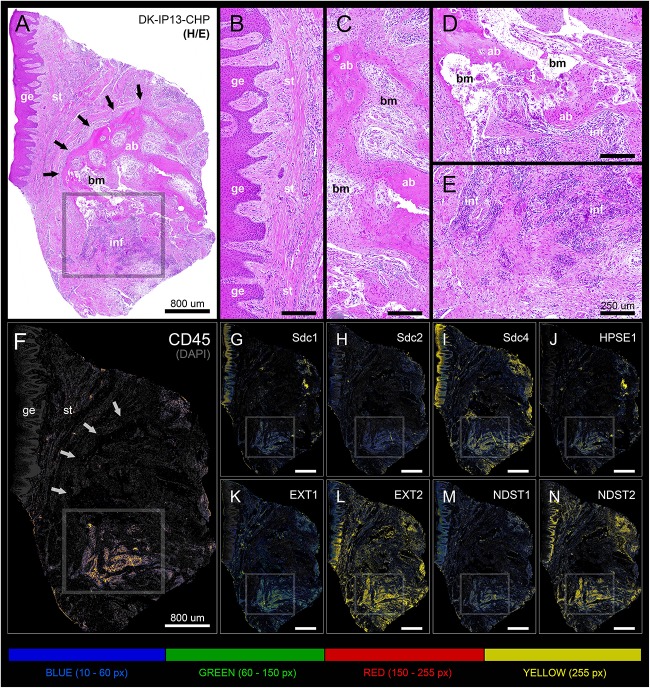
Panoramic H/E image **(A)** and panoramic multi-color heatmaps **(F–N)** of diseased gingiva (periodontitis sample DK-IP13-CHP) of expression domains of Sdcs **(G–I)**, HPSE1 **(J)**, EXTs **(K,L)**, NDSTs **(M,N)** and inflammatory marker CD45 **(F)** merged with DAPI nuclear staining (dark gray). Inflammatory infiltrate degrading alveolar bone is visible (framed areas) **(D,E)**. Subepithelial stroma between the gingival epithelium and intact lamellae of alveolar bone is mostly devoid of inflammatory infiltrate **(B)**. Bone marrow contains portions of fibrotic tissue **(C)**. All investigated factors are expressed in the area of inflammatory infiltrate **(G–N)** with variable intensity. Magnification: **(A–N)** ×10; *scale bars* – **(A,F–N)** 800 μm; **(B–E)** 250 μm. Designations : gingival epithelium (ge), subepithelial stroma (st), alveolar bone (ab), bone marrow (bm), inflammatory infiltrate (inf). Interface of subepithelial stroma and alveolar bone (black and white thick arrows). Intensity range increments: BLUE (10–60 px), GREEN (60–150 px), RED (150–255 px), YELLOW (255 px).

**FIGURE 6:**
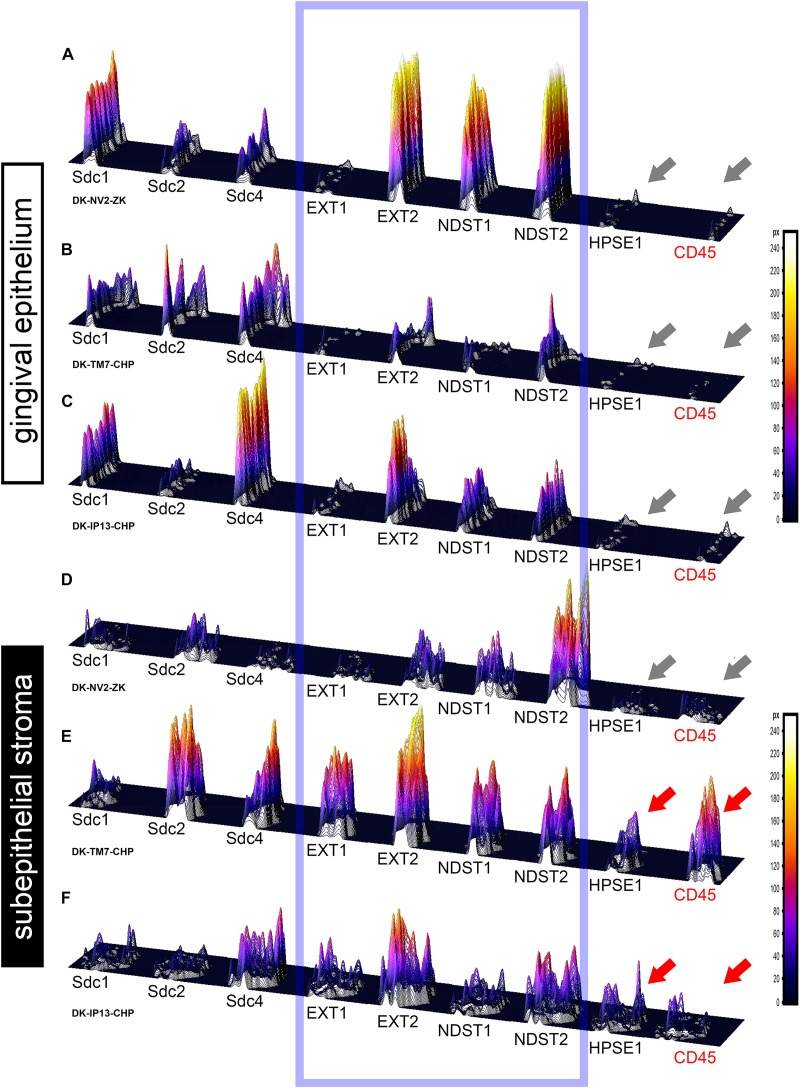
3D surface plots for multiple co-localization of expression of investigated factors in gingival epithelium **(A–C)** and subepithelial stroma **(D–F)** of healthy **(A,D)** (sample DK-NV2-ZK) and diseased gingiva **(B,C,E,F)** (samples DK-TM7-CHP, DK.IP13-CHP). The variable patterns of combinatorial expression of EXTs and NDSTs (framed area) might imply differences in HS composition between epithelial and stromal compartments within and between samples of healthy and diseased gingiva. Combinatorial expression of HPSE1 and CD45 (gray and red arrows) varies in healthy and diseased gingigiva, as well as in diseased gingiva samples with diffuse **(E)** and localized **(F)** inflammatory infiltrate, corresponding to the severity of inflammation. (Far right – color-graded intensity scales in px values).

### Expression of EXTs and NDSTs – IHC and Densitometry

In controls, the expression of EXT1 was very weak, whereas EXT2, NDST1, and NDST2 were expressed strongly in epithelial compartment and less intensely throughout the subepithelial stroma (Figures 3F–I). 2D plot profiles reveal decreasing mean expression intensity gradients for EXT2, NDST1, and NDST2 in oral-sulcular direction, and opposing increasing intensity gradients in marginal-apical direction (Figures 4C,I). In periodontitis group, the expression of EXTs and NDSTs was increased in subepithelial stroma significantly overlapping with expression domain of CD45 (Figures 3O–R). 2D plot profiles reveal markedly elevated mean intensity gradient of EXT1 which increases in sulcular and apical direction (Figures 4D,J). In contrast to controls, the expression patterns of EXTs and NDSTs (especially EXT1 and NDST1) displayed a lot of variations in samples from periodontitis group (Figures 3O–R, 5K–N), which could be related to varying histological features of stromal compartments, especially with regard to the extent and profile of inflammatory infiltrate. However, these deviations in EXTs and NDSTs molecular profiles are visible in both gingival epithelium (Figures 6A–C) and subepithelial stroma (Figures 6D–F).

### Statistical Analysis of Sdcs, HPSE1, EXTs, and NDSTs Expression Domains

**FIGURE 7:**
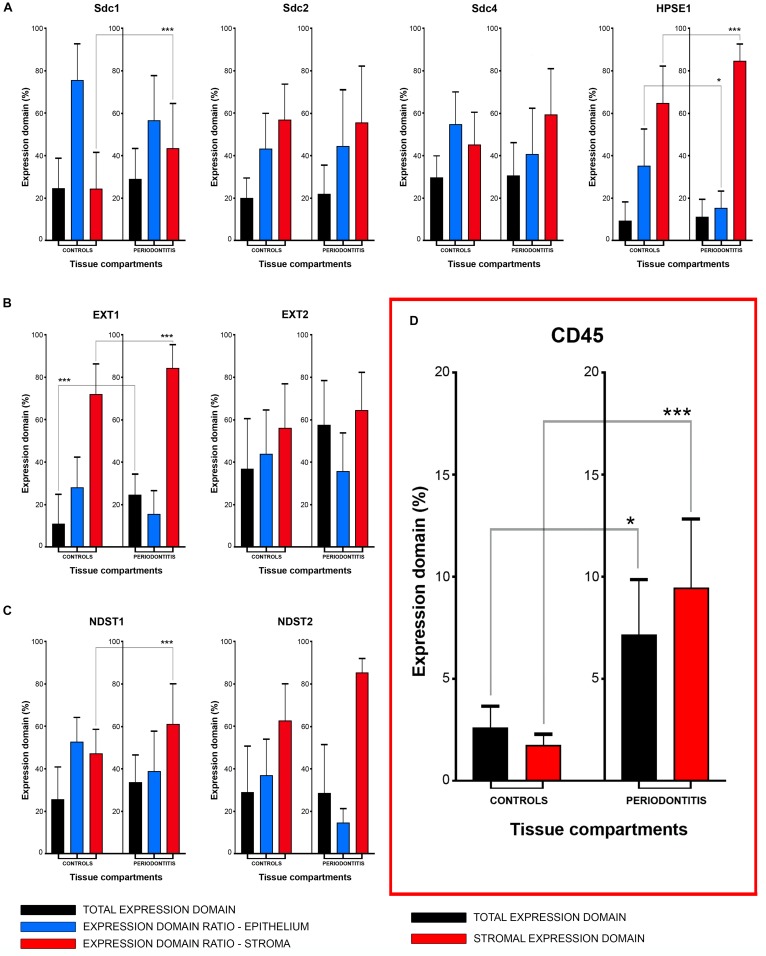
Mean group values of total expression domains (expressed in percentages as fractions of total section area) compared with epithelial and stromal expression domain ratios (expressed in percentages fractions of total expression domain) of investigated factors in control and periodontitis group **(A–C)**. The total availability of Sdcs and HPSE1 seems to be on comparable levels in controls and periodontitis, but their distribution (apart from Sdc2) in epithelial and stromal compartments of gingival tissue differs between two groups **(A)**. EXTs and NDST1 display similar pattern of changes in epithelial/stromal expression domain ratios in favor of stromal compartment **(B,C)**, which corresponds with increased presence of inflammatory infiltrate in periodontitis group (^∗∗∗^*p* = 0,003747) **(D)**. Level of significance was set at α = 0,01 (^∗∗∗^*p* < 0,01; ^∗^border values α = 0,05).

The initial comparison of group means for total expression domains of all investigated factors at level of significance α = 0,01 revealed that only EXT1 is differentially regulated in periodontitis (*p* = 4,5519 × 10^–6^) (Table 2). Thus, the additional comparison of group means of expression domains in epithelial and stromal compartments was also performed. No significant difference of investigated factors’ epithelial expression domains between controls and periodontitis group were found. However, the stromal expression domains of Sdc1 (*p* = 1,1033 × 10^–6^), HPSE1 (*p* = 0,0023), EXT1 (*p* = 1,2849 × 10^–7^), NDST1 (*p* = 0,000105), and CD45 (*p* = 0,003747) showed significant difference between controls and periodontitis group. The cumulative values of group means for total expression domains and ratios of epithelial and stromal expression domains were plotted on column graphs (Figure 7). Column graphs reveal that the total distribution of Sdcs and HPSE1 is almost equal in periodontitis group compared to controls, but the pattern of this distribution between epithelial and stromal compartments of gingival tissue (except for Sdc2) are quite different (Figure 7A). On the other hand, the distribution of EXTs and NDSTs seems to be more variable, both on the total level and between the epithelial and stromal compartments (Figures 7B,C). Additional aspects of control and periodontitis gingival tissue molecular profiling were revealed by correlating fractional values of investigated factors’ stromal expression domains with that of CD45 as dependent variable (Table 3). According to our model, Sdcs differentially correlate with the presence of inflammatory infiltrate. In controls, positive correlation was found for Sdc2, and negative correlation for Sdc4, whereas no correlation was found for Sdc1. In periodontitis group, both Sdc1 and Sdc2 were positively correlated, but no correlation was found for Sdc4. Interestingly, HPSE1, EXTs, and NDSTs displayed similar correlation pattern in control and periodontitis group – positive correlations were found for EXT1, EXT2 and NDST1. HPSE1 and NDST2 were correlated negatively with CD45.

**TABLE 2:** ANOVA analysis of total, epithelial, and stromal expression domains of investigated factors in control and periodontitis group.

**Factor**	**Tissue compartment**	***F-value***	**P-value**
Sdc1	TOTAL	*2,931137*	0,087518
	EPITHELIUM	*0,002173*	0,962838
	**STROMA^∗∗∗^**	***24,348604***	**1,103 × 10^–6^**
Sdc2	TOTAL	*0,813112*	0,367644
	EPITHELIUM	*0,183157*	0,668861
	STROMA	*0,000684*	0,979139
Sdc4	TOTAL	*0,148252*	0,700377
	EPITHELIUM	*0,493848*	0,482549
	STROMA	*2,705549*	0,100640
HPSE1	TOTAL	*1,049814*	0,306055
	**EPITHELIUM^∗^**	***4,965093***	**0,026316**
	**STROMA^∗∗∗^**	***9,320604***	**0,002389**
EXT1	**TOTAL^∗∗∗^**	***21,496702***	**4,551 × 10^–6^**
	EPITHELIUM	*2,476544*	0,116201
	**STROMA^∗∗∗^**	***28,727777***	**1,284 × 10^–7^**
EXT2	TOTAL	*0,098317*	0,753991
	EPITHELIUM	*0,284528*	0,593990
	STROMA	*2,461318*	0,117326
NDST1	TOTAL	*1,031306*	0,310353
	EPITHELIUM	*0,315713*	0,574451
	**STROMA^∗∗∗^**	***15,285767***	**0,000105**
NDST2	TOTAL	*0,247242*	0,619246
	EPITHELIUM	*1,896305*	0,169121
	STROMA	*1,445718*	0,229796
CD45	**TOTAL^∗^**	***4,461122***	**0,035180**
	**STROMA^∗∗∗^**	***8,483681***	**0,003747**

*Level of significance was set at α = 0,01 (^∗∗∗^*p* < 0,01; F_*crit*_ = 6,686878); (^∗^border values, level of significance α = 0,05).*

**TABLE 3:** Correlation of stromal expression of investigated factors with the stromal expression of inflammatory marker CD45 (dependent variable) in control and periodontitis group by multiple linear regression.

**Controls**		**Adj. R Square = 0,999970**		**Significance (α = 0,01)**	
		
**Factor**	**Coefficients**	***Std. Error***	***P*-value**	**Correlation**	**Factor**
CD45	0,001153	*3,4873E-05*	**8,69889 × 10^–91^**	**y-intercept**	**CD45**
Sdc1	0,001101	*0,000648*	0,090655	**/**	Sdc1
Sdc2	0,055181	*0,011347*	**2,10704 × 10^–6^**	**+**	**Sdc2**
Sdc4	−0,034054	*0,009169*	**0,000254**	−	**Sdc4**
HPSE1^∗^	−0,017389	*0,00798*	**0,03030448^∗^**	−	**HPSE1**
EXT1	0,040905	*0,004848*	**3,24401 × 10^–15^**	**+**	**EXT1**
EXT2	0,088969	*0,004871*	**4,18827 × 10^–47^**	**+**	**EXT2**
NDST1	0,072287	*0,006357*	**3,38384 × 10^–24^**	**+**	**NDST1**
NDST2	−0,124233	*0,008917*	**1,25435 × 10^–32^**	−	**NDST2**

**Periodontitis**		**Adj. R Square = 0,999842**		**Significance (α = 0,01)**	
		
**Factor**	**Coefficients**	***Std. Error***	***P*-value**	**Correlation**	**Factor**

CD45	0,007491	*0,000401*	**2,03439 × 10^–48^**	**y-intercept**	**CD45**
Sdc1	0,223765	*0,050875*	**1,64866 × 10^–5^**	**+**	**Sdc1**
Sdc2	0,038956	*0,014481*	**0,007649**	**+**	**Sdc2**
Sdc4	−0,304709	*0,196676*	0,122646	**/**	Sdc4
HPSE1^∗^	−0,546769	*0,267466*	**0,042033^∗^**	−	**HPSE1^∗^**
EXT1^∗^	0,081325	*0,035296*	**0,022084^∗^**	**+**	**EXT1^∗^**
EXT2	0,301984	*0,017352*	**2,94539 × 10^–44^**	**+**	**EXT2**
NDST1	0,295806	*0,116812*	**0,011977**	**+**	**NDST1^∗^**
NDST2	−0,319424	*0,082515*	**0,000140**	−	**NDST2**

*Level of significance was set at α = 0,01 (^∗∗∗^*p* < 0,01); (+ positive correlation, − negative correlation, / no correlation); (^∗^border values, level of significance α = 0,05).*

## Discussion

Periodontitis is a common degenerative disease initiated by the bacteria found in subgingival biofilm, which trigger host inflammatory response whose progression is ultimately responsible for the destruction of tooth supporting alveolar bone and soft tissues (periodontal tissue) resulting in tooth loss (Bosshardt and Lang, 2005; Pihlstrom et al., 2005). To date, significant effort has been invested in the research on the involvement of host cells and inflammatory mediators in regulation of inflammatory response in periodontitis (Silva et al., 2015). However, the role of HSPGs (including Sdcs) in pathogenesis of periodontitis is still poorly investigated despite the fact that there are numerous reports, which go almost two decades back, about the critical involvement of these factors in modulation of various aspects of inflammatory response. The data presented in this study clearly indicate that Sdcs display different expression profiles in healthy and diseased periodontal tissue which is in agreement with previous reports (Oksala et al., 1997; Manakil et al., 2001; Kotsovilis et al., 2010). This is further accompanied by the differences in expression profiles of enzymes involved in biosynthesis and modification of HS GAG side chains (EXTs, NDSTs, and HPSE1), which are comparable to a certain degree with those described in other chronic degenerative diseases such as osteoarthritis (Chanalaris et al., 2019). And finally, Sdcs and HS biosynthesis and modifying enzymes could be involved in modulation of inflammatory response in periodontitis, since their expression profiles display different correlation patterns with the presence of inflammatory infiltrate in healthy and diseased periodontal tissue. However, in order to interpret these findings in the context of complex pathogenesis of periodontal disease, the available experimental data about how Sdcs and HS-related enzymes are actually involved in regulation of inflammatory response, need to be presented in more detail.

### Structural Changes in Tissue Microenvironment Might Affect HS GAG-Related and Inherent Properties of Sdcs to Modulate Inflammatory Response in Periodontitis

Sdcs can interact with a wide variety of mediators of inflammation and through these interactions they affect different segments of inflammatory response such as chemokine/cytokine gradient formation, leukocyte recruitment, microbial attachment and entry, as well as the protease balance and activity in matrix remodeling during the resolution of inflammation and tissue healing (Teng et al., 2012; Bonnans et al., 2014). However, their roles in regulation of inflammatory response cannot simply be considered as either pro-inflammatory or anti-inflammatory, because there is ample evidence from experimental studies that individual Sdcs can play both roles depending on the type of disease-instigating agents and the tissues in which the noxious challenge is exerted. For example, according to experimental models of dermatitis, colitis and myocardial infarction, Sdc1 could be considered as a negative regulator of leukocyte recruitment because *Sdc1*^–/–^ knockout mice exhibit increased neutrophil adhesion and transendothelial migration, despite the fact that Sdcs are closely related to leukocyte recruitment because of their ability to bind chemokines and form chemokine gradients on endothelial surface of blood vessels (for that matter, the most well-studied connections are those between endothelial Sdc1, Sdc2, and CXC chemokine IL-8) (Mahtouk et al., 2007; Vanhoutte et al., 2007; Beauvais et al., 2009; Floer et al., 2010; Jung et al., 2016). Furthermore, the presence of Sdcs as intact cell surface HSPGs or soluble ECM effectors due to shedding of Sdcs core protein ectodomains can diversely affect the formation of gradients of inflammatory mediators, and thus the confinement, progression or attenuation of inflammatory response as nicely demonstrated on experimental knockout mouse models of allergic lung disease, bleomycin-induced acute lung injury and Gram-positive toxic shock (Li et al., 2002; Xu et al., 2005; Hayashida et al., 2009). Cell surface or shed Sdcs can also promote the pathogenesis of infectious diseases on multiple levels from facilitating microbial attachment and entry into host cells (cell surface Sdcs) to disruption of host immune response (shed Sdcs) as reported in studies on *Herpes simplex* virus or *Pseudomonas aeruginosa* infections, respectively (Bucior et al., 2010; Bacsa et al., 2011). While most of the described roles of Sdcs in regulation of inflammatory response can be strictly associated with their ability to carry HS GAG chains, it is possible that, to a certain degree, those roles might also be related to specific properties of individual Sdcs core proteins. Namely, the underlying molecular mechanisms of functional redundancy between Sdc1 and Sdc4 during prenatal development are unclear. Single *Sdc1^–/–^* or *Sdc4^–/–^* knockout animals develop normally, but as adults display similar phenotypes of impaired host immune response when challenged in inflammatory or wound-healing models (Stepp et al., 2015; Kero et al., 2018). Expression of Sdcs is reported to be very responsive to structural changes of tissue. However, it seems that during the inflammatory response these changes do not only affect the expression of Sdcs, but might also serve to unlock otherwise hidden inherent properties of individual Sdcs. The similar effect of tissue dynamics turning structural proteins into effective mediators of inflammatory response in chronic diseases has been described for many HSPGs and other ECM-related proteins (Rozario and DeSimone, 2010; Afratis et al., 2017). Accordingly, different expression profiles of Sdcs in healthy and diseased periodontal tissue reported here and in previous studies, might imply that perturbations in natural HS GAG-carrier capacity (and bio-availability of HS GAGS) does not need to be the only mechanism how Sdcs could modulate inflammatory response in periodontitis.

### Differential Expression of HS Biosynthesis and Degradation Enzymes Could Modulate the Inflammatory Response in Healthy and Diseased Periodontal Tissue

Since most of the roles of Sdcs in inflammation can be related to their ability to carry HS GAG chains which determine the HSPGs affinity for various ligands, it is reasonable to conclude that the course of inflammatory response should greatly depend on the biochemical properties of HS. This is corroborated by several studies on experimental animal models of allergic lung disease and diabetic nephropathy where the targeted endothelial ablation of genes encoding enzymes involved in HS biosynthesis resulted in both attenuation or severe disruption of inflammatory response (Ge et al., 2018; Talsma et al., 2018). HS is a linear polysaccharide chain comprised of repeating disaccharide units of glucosamine (GlcNAc) and glucuronic acid (GlcA) modified in a step-wise manner by addition of sulfate groups (NDSTs, sulfotransferases) during its biosynthesis, or post-biosynthetically by removal of sulfate groups at specific residues (desulfatases) and cleavage on small oligosaccharide fragments (HPSE1) (Zhang et al., 2016). While the total capacity of HS for ligands may depend upon its chain length (determined by EXTs polymerases), the affinity of HS is greatly influenced by the pattern and sequence of initial sulfation modifications (NDSTs). According to several reports, the roles of EXTs and NDSTs in the initial steps of HS biosynthesis are so intricately related that the variations in both HS chain length and structure must be viewed through prism of combinatorial actions of both EXTs and NDSTs (Esko and Selleck, 2002; Presto et al., 2008; Dagalv et al., 2015; Deligny et al., 2016). For example, altered activity of individual EXTs can also affect the pattern of HS sulfation modifications, whereas the altered activity of individual NDSTs can in turn affect the chain length and quantity of HS. Based on the data presented here, it is not possible to draw any definitive conclusions about the changes of quantity or composition of HS in healthy and diseased periodontal tissue. However, the differential expression profiles of EXT1, NDST1 and HPSE1 do imply that certain perturbations in stromal HS content and composition might occur in periodontitis and might be responsible for dysregulation of inflammatory response. Similar correlation profiles of EXTs and NDSTs with the presence of inflammatory infiltrate in healthy and diseased periodontal tissues are also intriguing, but more data about expression of other enzymes related to HS biosynthesis and modification are still needed in order to fully assess the potential significance of this finding. In spite of the statistically significant difference of expression of HPSE1 between healthy and diseased gingiva, negative correlation of expression of HPSE1 with the presence of inflammatory infiltrate in gingiva from both test and periodontitis group was a bit surprising. The increased activity of HPSE1 is considered as a hallmark of inflammatory sites in various tissues designating HPSE1 as a pro-inflammatory marker (Li and Vlodavsky, 2009). However, there are reports from studies on experimental animals that HPSE1 might also act as an anti-inflammatory agent in certain settings (Sanderson et al., 2017). Additionally, changes in expression of HPSE1 should not be definitively associated with the changes in HPSE1 enzymatic activity, because the commercial antibodies against HPSE1 do not differentiate between the inactive and active forms of HPSE1 (Kero et al., 2018). With regard to that, IHC profiling of gingival tissue with antibodies against specific epitopes of HS GAG side chains might prove useful.

Another difficulty about the analysis of expression profiles of HSPGs and HS-related enzymes is that these proteins are ubiquitously expressed in various tissues and as such require whole tissue section visualization, which fortunately today can be performed at high resolution. However, in order to fully utilize that, the quantification of IHC staining must also be performed at whole tissue section level, but in such a manner that the very shapes of tissue sections do not pose serious bias for subsequent statistical analysis. Here, this was achieved with regard to two attributes of IHC staining – the total area covered by staining (expression domain) and the distribution of intensity on a fixed ordinal scale (px values). The quantification of IHC staining gradients (i.e., the quantification of spatial distribution of staining) on a whole tissue section level still needs to be improved in order to obtain more detailed description of microenvironment in which the various cellular processes take place. This is of paramount importance, because the regulation of inflammatory response in periodontitis might also be driven by structural changes of microenvironment, whose molecular features are still insufficiently explored. Additional reason for continuing this line of research is provided by the experimental studies on application of HS-mimetics as potential therapeutic agents for regenerative treatment of periodontitis (Lallam-Laroye et al., 2006; Barritault et al., 2017).

## Conclusion

Of all investigated factors, the expression domains of Sdc1, HPSE1, EXT1, and NDST1 in gingival tissue displayed statistically significant differences between control and periodontitis group. The expressions of Sdcs in gingival tissue correlated differentially with the presence of inflammatory infiltrate in control and periodontitis group – in control group, no correlation was found for Sdc1, whereas Sdc2 correlated positively and Sdc4 correlated negatively; in periodontitis group both Sdc1 and Sdc2 correlated positively with the presence of inflammatory infiltrate, and no correlation was found for Sdc4. The expressions of HS biosynthesis (EXT1, EXT2, NDST1, and NDST2) and degradation enzymes (HPSE1) display similar correlation patterns with the presence of inflammatory infiltrate in gingival tissue from control and periodontitis group – EXT1, EXT2, and NDST1 correlate positively, whereas negative correlation was found for NDST2 and HPSE1. The analyzed histomorphometric parameters of gingival tissues were mostly found to be well-balanced between the control and periodontitis group with the exception of epithelial-stromal celularity ratio. Therefore, it might be suggested that the homeostasis in diseased gingival tissue is maintained to a significant degree, but the underlying compensatory mechanisms differ from those in healthy gingiva and could be related to the variable presence of inflammatory infiltrate and changes in expression profiles of Sdcs and HS biosynthesis and degradation enzymes. The findings presented here might also be viewed with regard to demographic risk factors for periodontitis such as age and smoking (Calsina et al., 2002; Helal et al., 2019). Namely, participants from the periodontitis group had higher mean age and were predominantly smokers in contrast to the participants from the control group (Table 1). Aging and smoking can affect immunity on multiple levels, but how these risk factors are exactly related to changes of HSPGs and HSPG-related factors’ molecular profiles during the inflammatory response in periodontitis still needs to be determined and, due to type of data and methods of analysis applied here, goes beyond the scope of this study.

## Data Availability Statement

All datasets generated for this study are included in the manuscript/supplementary files.

## Ethics Statement

Tissue procurement and processing were approved by the Ethical and Drug Committee of School of Medicine (University of Split) (Class: 003-08/17-03/0001, No: 2181-198-03-04-17-0043) and Ethical and Drug Committee of School of Dental Medicine (University of Zagreb) (No: 05-PA-15-6/2017) in accordance with Helsinki Declaration (Williams, 2008).

## Author Contributions

RDu assisted DK in the study design, performed IF staining, H/E and panoramic IF image acquisition and statistical analysis of the data, reviewed literature and wrote sections of the manuscript with MR (Introduction, Materials and Methods, Results, and Discussion), proofread the manuscript. MR assisted DK in the study design, devised protocol for patient screening and recruitment, collected tissue samples, participated in samples processing, interpreted data (clinical parameters and H/E staining), reviewed literature, wrote sections of the manuscript with RDu (Introduction, Materials and Methods, Results, and Discussion), proofread the manuscript. IP collected tissue samples, interpreted data (clinical parameters), wrote parts of the manuscript (Introduction, Materials and Methods, and Discussion), reviewed the literature, proofread the manuscript. NV participated in patient screening and recruitment, assisted in IF staining, reviewed part of the literature and proofread the manuscript. RDr participated in patient screening and recruitment, assisted in IF staining, reviewed part of the literature, and proofread the manuscript. KV supervised H/E and IF staining, reviewed part of the literature, interpreted data (H/E and IF staining), and proofread the manuscript. MS-B interpreted data (H/E and IF staining) and proofread the manuscript. DK designed the study, devised methods for image acquisition (H/E and IF panoramic images) and statistical analysis, performed selection of primary antibodies, provided theoretical background, interpreted data, reviewed literature, wrote and revised the manuscript.

## Conflict of Interest

The authors declare that the research was conducted in the absence of any commercial or financial relationships that could be construed as a potential conflict of interest.
